# Childbirth Experience Questionnaire (CEQ) in the Sri Lankan setting: translation, cultural adaptation and validation into the Sinhala language

**DOI:** 10.1186/s13104-020-05380-z

**Published:** 2020-11-13

**Authors:** Malitha Patabendige, Thilina S. Palihawadana, Rasika P. Herath, Prasantha S. Wijesinghe

**Affiliations:** 1 University Obstetrics Unit, North Colombo Teaching Hospital (NCTH), Ragama, Sri Lanka; 2 Department of Obstetrics and Gynaecology, Faculty of Medicine, University of Kelaniya, Ragama, Sri Lanka

**Keywords:** Childbirth experience questionnaire, Birth satisfaction, Low-resource settings

## Abstract

**Objective:**

To adapt the CEQ into Sri Lankan Sinhala cultural context and to determine the psychometric properties of CEQ. This would yield an opportunity to evaluate childbirth experience among Lankan population.

**Results:**

Out of 390, 226 (57.9%) postnatal mothers completed the CEQ after 1 month postpartum. Face validity and content validity were demonstrated with all participants stating that CEQ was easy to understand and complete. For reliability, internal consistency was acceptable for the overall score (0.85) and for all four domains in CEQ (0.65, 0.80, 0.70, 0.83 for “own capacity”, “professional support”, “perceived safety” and “participation”, respectively). A weighted kappa of 0.61–0.80 for all 22 items in CEQ demonstrated a good test–retest reliability. This Sri Lankan version showed fit statistics in line with standard recommendations in exploratory factor analysis. Women with spontaneous onset of labour (except for “professional support” in women with spontaneous onset of labour) and women with a normal birth showed significantly higher CEQ scores. However, oxytocin augmentation could not yield a difference in CEQ scores.

## Introduction

Childbirth Experience Questionnaire (CEQ) was derived and validated among first-time mothers in 2010 in Sweden [1]. It has been validated into several languages [2–6].

## Main text

CEQ measures four domains of the woman’s childbirth experience. Those are Own capacity, Professional support, Perceived safety and Participation, comprising of 22 items [2, 7]. CEQ is a self-administered tool to assess experience of women in different aspects of their labour and childbirth [7].

Women’s perceptions and experiences in childbirth are being barely evaluated in the Sri Lankan setting despite relatively better maternal and neonatal statistics in the region [8]. Every year 25–30 women commit suicide during pregnancy or within 1 year after birth in Sri Lanka [9]. Psychological morbidity among pregnant and postnatal mothers is an important area. Therefore, there is an urgent need to evaluate the quality of maternity care. CEQ seems to be the first tool validated in Sri Lanka aiming to assess childbirth experience. This would yield an opportunity to evaluate childbirth experience among Lankan population.

Objectives were to conduct a linguistic translation, cultural adaptation and to conduct a validation study assessing the psychometric properties of the Sinhala version of the CEQ.

### Method

#### Design and setting

A questionnaire validation study was carried out to translate and validate the CEQ from English to Sinhala at University Obstetrics Unit, North Colombo Teaching Hospital, Ragama, Sri Lanka. The original English language CEQ was translated into Sinhala language according to the standard validation principles as recommended by the World Health Organization (WHO) [10]. Cultural adaptation was further supported by the guidance given by Wild et al. [11]. Study protocol of this study has been published in *BMC Research Notes* Journal in 2019 [12].

#### Study population and sample size

Subjects to control ratio of 1:1 was taken using known-groups validation. Subjects were women who were nulliparous and undergoing operative births (forceps deliveries, vacuum deliveries, emergency caesarean deliveries). Controls were women who were nulliparous and undergoing normal deliveries. A minimum sample size of 315 was taken considering a 70% expected response rate and 10 times the observed variables [7, 11].

#### Inclusion and exclusion criteria

Women who were more than 16 years of age and who have undergone their first labour and childbirth experience in last 24–48 h (including women who were having a caesarean birth in their latent phase of labour and failed induction of labour) were included. Women who were sending from labour ward and undergoing emergency caesarean birth in last 24–48 h were also included.

Women with intrauterine fetal death or women with their babies were getting admitted to a special baby unit and those who had, a multiple pregnancy, an elective caesarean birth and women who were not educated up to General Certificate of Education (GCE) Ordinary Level were excluded.

#### Translation process

The linguistic translation process was carried out involving following basic steps [10]. Literal translation (forward translation) and adaptation of the English questionnaire into Sinhala lifestyle and cultural context were undertaken by two accredited bilingual native Sinhala speakers. Discussion with obstetricians, labour ward staff and patients as to whether the questionnaire measures what it was designed to measure and that clinically meaningful aspects were not lacking. With their comments, the Sinhala version of questionnaire was revised and rephrased according to Sri Lankan cultural and language context and compiled them into one Sinhala language version. Then, back-translated to English and this translator was blinded from the original English CEQ. All these translators have prior experience in the translation of Person Reported Outcomes (PRO) measures and they are permanent residents in Sri Lanka where the translation is to be used. Review of the back-translation was carried out by the CEQ team at the Sahlgrenska Academy, University of Gothenburg, Sweden. The final Sinhala CEQ version was produced after modifications, re-translation of necessary items to balance the discrepancies during translation process. Validation study was carried out using this final version (Additional file 1: CEQ-Sinhala).

#### Study instrument-Sinhala version of the CEQ

The CEQ consists of 22 items referring to the childbirth experience (Table 2). Women’s responses to 19 items in CEQ were rated on a 4-point Likert scale and remaining 3 items were evaluated by means of a visual analogue scale (VAS). These VAS scores are then converted into categorical variables facilitating interpretation and analysis; 0–40 = 1, 41–60 = 2, 61–80 = 3 and 81–100 = 4. As mentioned in Table 2, negatively worded items were reversed.

#### Data collection procedure

Eligible women were identified in the postnatal ward by the investigator and were invited to participate. Two copies of CEQ with two stamped-envelopes were given to each mother and were asked to fill each in 1 month postnatal and 6 weeks postnatal. Reminders were sent after 1 month and 6 weeks as a phone call or a post card.

#### Data analysis

Basic demographic characteristics of the data were analysed to see any deviations. Continuous variables were presented as means and standard deviations. Pearson Chi Square test was applied to any difference between categorical variables. Mann–Whitney U test was applied to compare any skewed continuous variables. p value < 0.05 was considered as statistically significant. Face validity, content validity, reliability, construct validity, discriminant validity, test–retest reliability were assessed.

#### Ethical considerations

Ethical aspects of this study were reviewed and approved by the Ethical Review Committee, Faculty of Medicine, University of Kelaniya, Ragama, Sri Lanka.

### Results

#### Participant characteristics

Of the 390 eligible post-partum women who met the inclusion criteria, 226 (57.9% response rate) women had completed and returned the first CEQ between one and four months after birth. Twelve questionnaires were incomplete and excluded from analysis yielding a final sample of 214. Women with higher level of education (G.C.E. Advanced Level passed and above) showed a significantly better response rate compared to women educated up to G.C.E. Ordinary Level (p value < 0.05). Unemployed women also showed a significantly better response rate compared to employed women (p value < 0.05). However, level of income, mode of delivery and type of onset of labour did not show any significant impact on the response rate. Table 1 summarizes basic demographic and clinical characteristics.

**Table 1:** Basic demographic and clinical characteristics of the study participants

Characteristics of the study participants (n = 214)	
Variables	n (%)
Education	
G.C.E. O/L passed	105 (49.1)
G.C.E. A/L passed	84 (39.3)
Diploma/University degree	25 (11.6)
Employed	64 (30.0)
Monthly income in USD	
Less than 110	2 (0.9)
Between 110 and 330	173 (80.8)
More than 330	39 (18.2)
Maternal age in years, mean (SD)	28.7 (4.5)
Gestational age in weeks, mean (SD)	39.5 (1.2)
Onset of labour	
Spontaneous	121 (56.5)
More than 12 h of labour duration	2 (0.9)
Type of birth	
Normal birth	162 (75.7)
Forceps birth	5 (2.3)
Vacuum birth	10 (4.7)
Caesarean birth	37 (17.3)
Perineal status after birth	
Third/fourth degree tears	2 (0.9)

G.C.E. O/L: General Certificate of Education Ordinary Level; G.C.E. A/L: General Certificate of Education Advanced Level; USD: United States Doller; SD: Standard Deviation

#### Face validity

In a face-to-face interview, 25 postpartum women revealed that items were easy to understand and to complete in 15 min, giving a good face validity.

#### Content validity

Labour ward staff (n = 7) and the obstetric expert team mentioned that the questionnaire was easy to understand. According to their responses some changes were made. “*Midwives devoted enough time to my relative/relatives*” were used in Sinhala version instead of “*My midwife also devoted enough time to my partner*” in the original version. “*My midwives*” were used instead “*my midwife*”.

#### Reliability

Sinhala version yielded a good internal consistency as measured by Cronbach’s alpha for respective domains (Own capacity 0.65, Professional support 0.80, Perceived safety 0.70, Participation 0.83) and the total scale (0.85). Interclass correlation coefficient was 0.85 with a p value < 0.0001. After deleting “each” item, the reliability of the questionnaire reduced.

Ninety women returned the second CEQ and the quadratic weighted index of agreement (weighted Kappa) for each of the 22 items has presented in Table 2. All 22 items were found to have a considerable agreement between the scores gained at 1 month and then 6 weeks postpartum respectively.

**Table 2:** Description of 22 items and scores in Childbirth experience questionnaire (CEQ)

Item in CEQ	Total sample in each item (n = 214)	Mean Score (SD)	Weighted Kappa scores between 1st and 2nd CEQ
1. Labour progressed as I had expected	214	3.0 (0.9)	0.83
2. I felt strong	214	3.4 (0.6)	0.66
3. I felt scared	214	2.0 (0.9)	0.74
4. I felt strong capable	214	3.5 (0.6)	0.76
5. I felt strong tired^b^	214	3.6 (0.7)	0.71
6. I felt happy	214	3.6 (0.7)	0.74
7. I have many positive memories from the labour process	214	3.4 (0.9)	0.69
8. I have many negative memories from the labour process^b^	214	3.2 (1.0)	0.67
9. Some of my memories from the labour process make me feel depressed^b^	214	3.4 (0.9)	0.68
10. I felt I could choose whether I should be up and moving or lie down	21	2.3 (1.1	0.6
11. I felt I could choose the delivery position	214	2.6 (1.1)	0.72
12. I felt I could choose which pain relief method to use	214	2.5 (1.1)	0.70
13. Midwives devoted enough time to me	214	2.9 (0.9)	0.67
14. Midwives also devoted enough time to my relative/relatives	214	2.9 (0.9)	0.71
15. Midwives kept me informed about what was happening during labour and birth	214	3.3 (0.8)	0.82
16. Midwives understood my needs.	214	3.4 (0.8)	0.77
17. I felt very well taken care of by the midwives	214	3.6 (0.7)	0.66
18. My impression of the medical competence made me feel secures	214	3.6 (0.6)	0.65
19. I felt that I handled the situation well.	214	3.4 (0.7)	0.71
20. Experienced level of labour pain, VAS^a,b^	214	1.6 (0.9)	0.65
21. Experienced level of control, VAS^a^	214	2.9 (1.1)	0.66
22. Experienced level of sense of security, VAS^a^	214	3.5 (0.9)	0.68

VAS: Visual Analogue Scale; CEQ: Childbirth Experience Questionnaire

^a^VAS-scales scores were recoded to categorical values, 0–40 = 1, 41–60 = 2, 61–80 = 3 and 81–100 = 4

^b^ Ratings of negatively worded statements are reversed

#### Construct validity

Total 22 items were initially entered in the exploratory factor analysis. The Kaiser–Meyer–Olkin (KMO) value was 0.82, and Bartlett’s Test of Sphericity reached statistical significance (p < 0.001) supporting the factorability of the correlation matrix. The principle component analysis revealed the presence of six factors with Eigen value exceeding 1, explaining cumulative variance of 63.5% with varimax rotation in the rotated component matrix and scree plot (Additional file 2: Fig. S1).

#### Discriminant validity

Known-group validation was applied to assess discriminant validity and details have been summarized in Table 3. Only two women had labour duration more than 12 h and therefore labour duration was not taken into account. Women with spontaneous onset of labour and normal birth groups obtained significantly higher CEQ scores, when compared to women whose labour had been induced and who had an instrumental birth, respectively. Women who had a spontaneous onset of labour obtained statistically significantly higher scores (p value < 0.05) in each of the following subscales: “own capacity”, “perceived safety”, “participation” and overall CEQ score, except for “professional support”. Women who had a normal birth obtained statistically significant higher scores than mothers with an instrumental birth, for all four subscales and overall score.

**Table 3:** Difference between subscale scores and overall score of the CEQ in contrast to known groups-Discriminant validity

Known-groups	CEQ score				
	Own capacity	Professional support	Perceived safety	Participation	Overall CEQ score
Spontaneous onset of labour n = 121	2.96 (0.42)	3.37 (0.52)	3.26 (0.54)	2.57 (0.95)	3.08 (0.42)
Induction of labour n = 93	2.87 (0.45)	3.33 (0.66)	3.09 (0.52)	2.35 (0.92)	2.96 (0.43)
p value^a^	0.04	0.86	0.009	0.04	0.01
Effect size	0.14	0.012	0.18	0.11	0.17
No oxytocin augmentation n = 137	2.95 (0.36)	3.34 (0.57)	3.21 (0.49)	2.39 (0.95)	3.03 (0.37)
Oxytocin augmentation n = 77	2.87 (0.55)	3.36 (0.62)	3.15 (0.64)	2.63 (0.90)	3.02 (0.52)
p-value^a^	0.49	0.54	0.98	0.07	0.59
Effect size	0.05	0.04	0.001	0.12	0.04
Normal birth n = 162	3.05 (0.36)	3.49 (0.55)	3.29 (0.40)	2.56 (0.93)	3.15 (0.33)
Operative birth n = 52	2.69 (0.54)	3.17 (0.68)	3.03 (0.65)	2.26 (0.84)	2.83 (0.53)
p-value^a^	0.001	0.01	0.04	0.01	0.005
Effect size	0.34	0.27	0.19	0.18	0.3

Data has presented as mean (SD)

CEQ: Childbirth Experience Questionnaire

^a^ Mann–Whitney U-test was used to compute p-values as the scores were not parametrically distributed. Operative birth consists of caesarean and instrumental births. Overall score for the CEQ is the mean score of its 4 subscales

### Discussion

This study describes a successful transcultural adaptation of the CEQ into Sinhala language in Sri Lankan context. Sinhala version of the CEQ (CEQ-Sinhala) has shown to be a valid and reliable tool to assess childbirth experience among Sinhala speaking Sri Lankan women. To improve maternal healthcare services in a society, mothers’ opinions and expectations should be taken into account [13, 14]. Larkin et al, further elaborates that identification of the core attributes of the labour/childbirth experience might yield a framework for future planning and considerations [15]. These type of tools can help to improve maternal health services in order to tailor women’s expectations in labour and childbirth process. Disrespectful maternity care is prevalent in many settings throughout the globe, particularly in under-resource settings [16, 17]. Latest WHO intrapartum care recommendations targeting a positive childbirth experience elaborate this furthermore [14].

Our study could not demonstrate any significant difference in CEQ scores between augmentation of labour with oxytocin versus no oxytocin augmentation. It worthwhile to mention that oxytocin infusions are commonly used in Sri Lankan labour wards for the majority of women in labour. We had only two-third/fourth degree perineal tears and therefore it was not included in construct validity. Although original CEQ was developed for mothers after their first childbirth, Spanish CEQ validation study found a novel thing that CEQ can also be used for multiparous women [2]. They have also showed that the Spanish adaptation could discriminate birth experience between primiparous and multiparous women as a test for construct validity [2]. We found the lowest mean CEQ score as 1.6 for the item 20 (“Experienced level of labour pain”). It also had a good stability (kappa 0.66). This signals that more emphasis has to be paid on labour analgesia.

In Sri Lanka, labour companion (*doula*) is allowed in some settings only and in most of the labour wards, relatives are staying outside the labour ward. Partner is not allowed in almost all the labour wards. However, they are free to contact labour ward staff and clarify any information about labouring woman. This a point for cultural adaptation and therefore, we have adapted the item as “*Midwives devoted enough time to my relative/relatives*” instead of “*My midwife also devoted enough time to my partner*” as in the original version. However, the authors expected that new generation of mothers are educated with different views and ground level of birth culture assumed to be evolving as in many other countries. As above, we have adapted the use of “*Midwives*” to our setting as we have common labour wards instead of “*My midwife*”. The CEQ is supposed to measure women’s childbirth experience, not the birth satisfaction hence these cultural adaptation may yield a more practical tool to Sri Lanka. All these changes were made in view of a better cultural adaptation to the Sri Lankan version and guided by the Wild et al. [11].

## Conclusion

Sinhala translation of the CEQ (CEQ-Sinhala) is a reliable, culturally acceptable and valid tool with well-preserved psychometric properties to assess childbirth experience among Sinhala speaking women in Sri Lanka.

## Limitations

Criterion validity could not be computed due to unavailability of a ‘gold standard’ assessment of childbirth experience in Sri Lanka. Time lapses between childbirth and questionnaire could possibly give negative influence on birth experience.

## Supplementary information

**Additional file 1.** Sinhala version of the Childbirth Experience Questionnaire (CEQ-Sinhala).

**Additional file 2: Figure S1.** Scree plot of factor extracting in Sri Lankan version of Childbirth Experience Questionnaire with principal component analysis.

## Data Availability

Datasets generated from this study will be available from the corresponding author (MP) upon a reasonable request.

## Abbreviations

CEQ: Childbirth Experience Questionnaire
LMIC: Low-middle income countries
WHO: World Health Organization
VAS: Visual analogue scale
G.C.E. O/L: General Certificate of Education Ordinary Level
G.C.E. A/L: General Certificate of Education Advanced Level
USD: United States Doller
SD: Standard deviation
