# Supplementary material for: Childbirth Experience Questionnaire (CEQ) in the Sri Lankan setting: translation, cultural adaptation and validation into the Sinhala language
Source: BMC Res Notes. 2020 Nov 13;13:534. doi: 10.1186/s13104-020-05380-z (PMC7666445; doi:10.1186/s13104-020-05380-z)
Supplement: Supplementary file 1 — Additional file 1. Sinhala version of the Childbirth Experience Questionnaire (CEQ-Sinhala). [file 13104_2020_5380_MOESM1_ESM.pdf]

දරු උපතේදී ලද අත්දැකීම් පිළිබඳ ප්‍රශ්නාවලිය.

ආදරණීය නවක මව්වරුනි,

දරු ප්‍රසූතිය පිළිබඳ සත්කාර සේවයේ එක් ප්‍රධාන ඉලක්කයක් වනුයේ මවට දරු උපත පිළිබඳ සුභවාදී අත්දැකිමක් ලබාදීම තහවුරු කිරීමයි. මෙම ප්‍රශ්නාවලියෙහි අරමුණ වනුයේ ඔබ දරු උපතේදී අත්දැකීම් ලැබූ ආකාරය අධ්‍යයනය කිරීමයි. ඔබගේ පිළිතුරු දරු උපත පිළිබඳ සත්කාර සේවය විශ්ලේෂණය සඳහා යොදා ගැනේ. ඔබ සියළුම ප්‍රශ්න වලට පිළිතුරු සැපයීම ඉතා වැදගත්වේ.

ඔබගේ අත්දැකීම් ලකුණු කළ හැකි ආකාර 2 කි. එනම් අදාළ කොටුව තුල කතිරයක් යෙදීමෙන් හෝ ඉර මත සලකුණු කිරීම මඟිනි.

උදාහරණ:

1.

- මම දිනපතා පළතුරු කමි.

සම්පූර්ණයෙන්ම එකඟ වෙමි	බොහෝ දුරට එකඟ වෙමි	බොහෝ දුරට එකඟ නොවෙමි	කිසිසෙත්ම එකඟ නොවෙමි
☐	☒	☐	☐

2.

- ඔබ ඇපල් වලට කොච්චර කැමතිද?

කිසිසේත් කැමති නැත. | මගේ ප්‍රියතම පළතුර

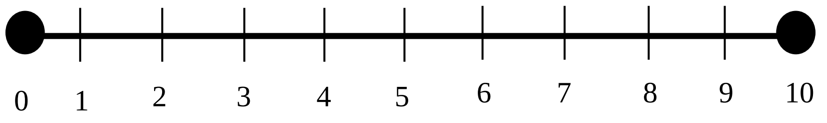

ප්‍රශ්නාවලිය මිලඟ පිටුවේ සිට ආරම්භවේ.

ඔබගේ සහභාගිත්වයට ස්තුතියි.

1. ප්‍රසූතිය හා දරු උපත මා බලාපොරොත්තු වූ ආකාරයටම සිදුවුනි.

සම්පූර්ණයෙන්ම එකඟ වෙමි	බොහෝ දුරට එකඟ වෙමි	බොහෝ දුරට එකඟ නොවෙමි	කිසිසෙත්ම එකඟ නොවෙමි
☐	☐	☐	☐

2. ප්‍රසූතියේදී හා දරු උපතේදී මට දිරිමත් බවක් දැනුණි.

සම්පූර්ණයෙන්ම එකඟ වෙමි	බොහෝ දුරට එකඟ වෙමි	බොහෝ දුරට එකඟ නොවෙමි	කිසිසෙත්ම එකඟ නොවෙමි
☐	☐	☐	☐

3. ප්‍රසූතියේදී හා දරු උපතේදී මට බියක් දැනුණි.

සම්පූර්ණයෙන්ම එකඟ වෙමි	බොහෝ දුරට එකඟ වෙමි	බොහෝ දුරට එකඟ නොවෙමි	කිසිසෙත්ම එකඟ නොවෙමි
☐	☐	☐	☐

4. ප්‍රසූතියේදී හා දරු උපතේදී මට එය කළ හැකි යැයි හැඟුනි.

සම්පූර්ණයෙන්ම එකඟ වෙමි	බොහෝ දුරට එකඟ වෙමි	බොහෝ දුරට එකඟ නොවෙමි	කිසිසෙත්ම එකඟ නොවෙමි
☐	☐	☐	☐

5. ප්‍රසූතියේදී හා දරු උපතේදී මා වෙහෙසට පත්වුණි.

සම්පූර්ණයෙන්ම එකඟ වෙමි	බොහෝ දුරට එකඟ වෙමි	බොහෝ දුරට එකඟ නොවෙමි	කිසිසෙත්ම එකඟ නොවෙමි
☐	☐	☐	☐

6. ප්‍රසූතියේදී හා දරු උපතේදී මා සතුටට පත්වුණි.

සම්පූර්ණයෙන්ම එකඟ වෙමි	බොහෝ දුරට එකඟ වෙමි	බොහෝ දුරට එකඟ නොවෙමි	කිසිසෙත්ම එකඟ නොවෙමි
☐	☐	☐	☐

7. මට දරු උපත පිළිබඳ බොහෝ ප්‍රසන්න මතකයන් ඇත.

සම්පූර්ණයෙන්ම එකඟ වෙමි	බොහෝ දුරට එකඟ වෙමි	බොහෝ දුරට එකඟ නොවෙමි	කිසිසෙත්ම එකඟ නොවෙමි
☐	☐	☐	☐

8. මට දරු උපත පිළිබඳ බොහෝ අප්‍රසන්න මතකයන්ද ඇත.

සම්පූර්ණයෙන්ම එකඟ වෙමි	බොහෝ දුරට එකඟ වෙමි	බොහෝ දුරට එකඟ නොවෙමි	කිසිසෙත්ම එකඟ නොවෙමි
☐	☐	☐	☐

9. මාගේ දරු උපත පිළිබඳ මතකයන් මා හට කළකිරුණු ස්වභාවයක් ඇති කරයි.

සම්පූර්ණයෙන්ම එකඟ වෙමි	බොහෝ දුරට එකඟ වෙමි	බොහෝ දුරට එකඟ නොවෙමි	කිසිසෙත්ම එකඟ නොවෙමි
☐	☐	☐	☐

10. දරු ප්‍රසූතිය ආසන්න වනතෙක් මා කැමැති ඉරියව්වකින් සිටීමට (ඉඳගෙන, සිටගෙන, වැතිර හෝ ඇවිදිමින්) මා හට අවස්ථාවක් ලබා දුන්නා නම් හොඳයි කියා හැඟුණි.

සම්පූර්ණයෙන්ම එකඟ වෙමි	බොහෝ දුරට එකඟ වෙමි	බොහෝ දුරට එකඟ නොවෙමි	කිසිසෙත්ම එකඟ නොවෙමි
☐	☐	☐	☐

11. දරුවා බිහිවෙන අවස්ථාවේදී එය පහසු වීම සඳහා මා සිටිය යුතු ඉරියව්ව තීරණය කිරීමේදී මා හටද අවස්ථාවක් ලබා දුන්නා නම් හොඳයි කියා හැඟුණි.

සම්පූර්ණයෙන්ම එකඟ වෙමි	බොහෝ දුරට එකඟ වෙමි	බොහෝ දුරට එකඟ නොවෙමි	කිසිසෙත්ම එකඟ නොවෙමි
☐	☐	☐	☐

12. ප්‍රසූත වේදනාව අවම කරගැනීමට වේදනා නාශක ක්‍රම තෝරා ගැනීමේදී මා හටද අවස්ථාවක් ලබා දුන්නා නම් හොඳයි කියා හැඟුණි.

සම්පූර්ණයෙන්ම එකඟ වෙමි	බොහෝ දුරට එකඟ වෙමි	බොහෝ දුරට එකඟ නොවෙමි	කිසිසෙත්ම එකඟ නොවෙමි
☐	☐	☐	☐

13. ප්‍රසූත සහායිකාවන් මට අවශ්‍ය තරම් සහයෝගය ලබා දුන්නෝය.

සම්පූර්ණයෙන්ම එකඟ වෙමි	බොහෝ දුරට එකඟ වෙමි	බොහෝ දුරට එකඟ නොවෙමි	කිසිසෙත්ම එකඟ නොවෙමි
☐	☐	☐	☐

14. ප්‍රසූත සහායිකාවන් මාගේ ඥාතින්ට/ඥාතියාට අවශ්‍ය තරම් සහයෝගය ලබා දුන්නෝය.

සම්පූර්ණයෙන්ම එකඟ වෙමි	බොහෝ දුරට එකඟ වෙමි	බොහෝ දුරට එකඟ නොවෙමි	කිසිසෙත්ම එකඟ නොවෙමි
☐	☐	☐	☐

15. ප්‍රසූත සහායිකාවන් දරු ප්‍රසූතිය හා උපත අතරතුර එහි කුමක් සිදුවෙමින් පවතිනවාද යන්න පිළිබඳ නිරන්තරයෙන් මාව දැනුවත් කර තැබුවෝය.

සම්පූර්ණයෙන්ම එකඟ වෙමි	බොහෝ දුරට එකඟ වෙමි	බොහෝ දුරට එකඟ නොවෙමි	කිසිසෙත්ම එකඟ නොවෙමි
☐	☐	☐	☐

16. දරු උපතේදී ප්‍රසූත සහායිකාවන් මාගේ අවශ්‍යතාවන් හඳුනා ගත්තෝය.

සම්පූර්ණයෙන්ම එකඟ වෙමි	බොහෝ දුරට එකඟ වෙමි	බොහෝ දුරට එකඟ නොවෙමි	කිසිසෙත්ම එකඟ නොවෙමි
☐	☐	☐	☐

17. ප්‍රසූත සහායිකාවන් මාව හොඳින් රැක බලාගත් බවක් මට දැනුණි.

සම්පූර්ණයෙන්ම එකඟ වෙමි	බොහෝ දුරට එකඟ වෙමි	බොහෝ දුරට එකඟ නොවෙමි	කිසිසෙත්ම එකඟ නොවෙමි
☐	☐	☐	☐

18. කණ්ඩායමේ වෛද්‍යමය දක්ෂතාවන් පිළිබඳ මා තුළ තිබූ විශ්වාසය නිසා මා හට ආරක්ෂාකාරී බවක් හැඟුණි.

සම්පූර්ණයෙන්ම එකඟ වෙමි	බොහෝ දුරට එකඟ වෙමි	බොහෝ දුරට එකඟ නොවෙමි	කිසිසෙත්ම එකඟ නොවෙමි
☐	☐	☐	☐

19. මම දරු ප්‍රසූතිය හොඳින් හැසිරවුවා යැයි මට හැඟුණි.

සම්පූර්ණයෙන්ම එකඟ වෙමි	බොහෝ දුරට එකඟ වෙමි	බොහෝ දුරට එකඟ නොවෙමි	කිසිසෙත්ම එකඟ නොවෙමි
☐	☐	☐	☐

20. සමස්ථයක් ලෙස දරු උපත කොපමණ වේදනාකාරී ලෙසට ඔබට හැඟුණේද?

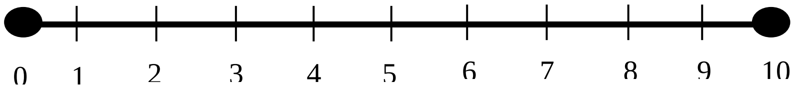

වේදනාවක් නැත. කිවහැකි උපරිම වේදනාව

21. සමස්ථයක් ලෙස දරු උපත අතරතුරදී ඔබට කොතරම් පාලනයක් තිබුණාදැයි ඔබට හැඟුණේද?

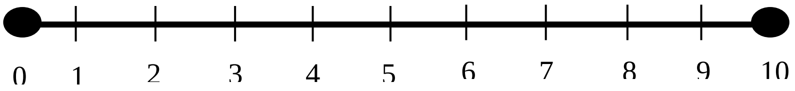

පාලනයක් නැත. සම්පූර්ණ පාලනයක් තිබිණි

22. සමස්ථයක් ලෙස දරු උපත අතරතුරදී ඔබට කොතරම් ආරක්ෂාකාරී බවක් දැනුණේද?

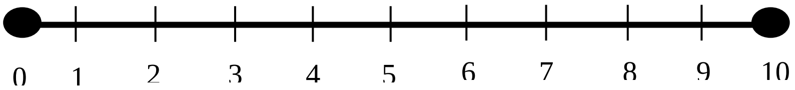

කිසිසේත්ම ආරක්ෂාකාරී බවක් නැත සම්පූර්ණයෙන්ම ආරක්ෂාකාරීය

අමතර අදහස්

ඔබගේ අදහස් දැක්වීමට ස්තුතියි !
