# Supplementary figures and images for: Childbirth Experience Questionnaire (CEQ) in the Sri Lankan setting: translation, cultural adaptation and validation into the Sinhala language
Source: BMC Res Notes. 2020 Nov 13;13:534. doi: 10.1186/s13104-020-05380-z (PMC7666445; doi:10.1186/s13104-020-05380-z)

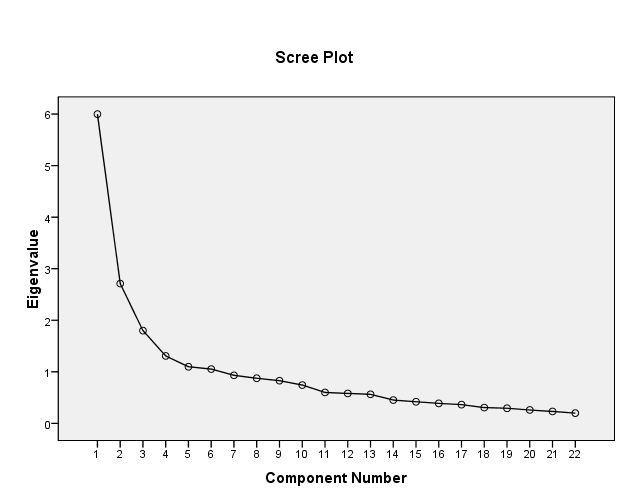

Supplement: Supplementary file 2 — Additional file 2: Figure S1. Scree plot of factor extracting in Sri Lankan version of Childbirth Experience Questionnaire with principal component analysis. [file 13104_2020_5380_MOESM2_ESM.png]
